# Influence of Germline *BRCA* Genotype on the Survival of Patients with Triple-Negative Breast Cancer

**DOI:** 10.1158/2767-9764.CRC-21-0099

**Published:** 2021-12-08

**Authors:** Cynthia Villarreal-Garza, Ana S. Ferrigno, Alejandro Aranda-Gutierrez, Paul H. Frankel, Nora H. Ruel, Alan Fonseca, Steven Narod, Yanin Chavarri-Guerra, Erika Sifuentes, Maria Cristina Magallanes-Hoyos, Josef Herzog, Danielle Castillo, Rosa M. Alvarez-Gomez, Alejandro Mohar-Betancourt, Jeffrey N. Weitzel

**Affiliations:** 1 Breast Cancer Center, Hospital Zambrano Hellion TecSalud, Tecnologico de Monterrey, San Pedro Garza Garcia, Nuevo Leon, Mexico.; 2 Instituto Nacional de Cancerología, Mexico City, Mexico.; 3 Instituto Nacional de Ciencias Médicas y Nutrición Salvador Zubirán, Mexico City, Mexico.; 4 City of Hope Cancer Center, Duarte, California.; 5 Women's College Research Institute, University of Toronto, Toronto, Ontario, Canada.; 6 Latin American School of Oncology, Sierra Madre, California.

## Abstract

The presence of *BRCA* pathogenic variants (PV) in triple-negative breast cancer (TNBC) is associated with a distinctive genomic profile that makes the tumor particularly susceptible to DNA-damaging treatments. However, patients with *BRCA* PVs can develop treatment resistance through the appearance of reversion mutations and restored *BRCA* expression. As copy-number variants (CNV) could be less susceptible to reversion mutations than point mutations, we hypothesize that carriers of *BRCA* CNVs may have improved survival after treatment compared with carriers of other *BRCA* PVs or *BRCA* wild-type. Women diagnosed with stage I–III TNBC at ≤50 years at a cancer center in Mexico City were screened for *BRCA* PVs using a recurrent PV assay (HISPANEL; 77% sensitivity). Recurrence-free survival (RFS) and overall survival (OS) were compared according to the mutational status. Among 180 women, 17 (9%) were carriers of *BRCA1* ex9–12del CNVs and 26 (14%) of other *BRCA* PVs. RFS at ten years for the whole cohort was 79.2% [95% confidence interval (CI), 72.3–84.6], with no significant differences according to mutational status. 10-year OS for the entire cohort was 85.3% (95% CI, 78.7–90.0), with *BRCA* CNV carriers demonstrating numerically superior OS rates other PV carriers and noncarriers (100% vs. 78.6% and 84.7%; log-rank *P* = 0.037 and *P =* 0.051, respectively). This study suggests that *BRCA1* ex9–12del CNV carriers with TNBC may have a better OS, and supports the hypothesis that the genotype of *BRCA* PVs may influence survival by limiting treatment resistance mediated by reversion mutations among CNV carriers.

**Significance:**

Large CNV *BRCA* carriers in a cohort of young Mexican patients with TNBC had superior OS rates than carriers of other *BRCA* pathogenic variants (i.e., small indels or point mutations). We hypothesize that this is due to the resistance of CNVs to reversion mutations mediating resistance to therapy. If validated, these findings have important prognostic and clinical treatment implications for *BRCA*-associated breast cancers.

## Introduction

Breast cancer is the most frequently diagnosed malignancy and the leading cause of cancer-related death in females (1). It is estimated that 5% to 10% of breast cancer cases are related to inherited predisposition genes, with germline *BRCA1* and *BRCA2* pathogenic variants (*BRCA* PV) being the most common (2, 3). The presence of these germline PVs can have an important effect on the characteristics of breast tumors. *BRCA1*-mutated breast cancers tend to have a high histologic grade and be classified as triple-negative breast cancer (TNBC), while tumors associated with *BRCA2* PVs are associated with estrogen receptor (ER)-positive and HER2-negative subtype, but with a tendency toward a higher proportion of TNBC with increasing age (4).

Despite the effects of *BRCA* PVs on the clinicopathologic features of breast tumors, the prognostic effect of these PVs is less clear (5–7). In general, it appears that no significant difference exists in overall survival (OS) in comparison to phenotypically similar tumors of noncarriers of *BRCA* PVs, except for a tendency toward a worse OS in ER-positive *BRCA*-associated tumors (8, 9). Recent studies of TNBC did not reveal significant correlation between *BRCA* status and recurrence-free survival (RFS) or OS (10–12). Nevertheless, the Prospective Outcomes in Sporadic versus Hereditary breast cancer (POSH) study reported that *BRCA* PV carriers with young-onset TNBC (defined as women aged <40 years at diagnosis) may have a survival advantage in the first 2 years after detection, although this was not statistically significant after 5 years (12). A possible explanation for the observed disparities in previous literature is a genotype–phenotype correlation, meaning that not all *BRCA* PVs have the same impact on breast cancer treatment response and survival outcomes (13, 14).

A key function of the *BRCA* tumor suppressor genes is to maintain genomic stability through homologous recombination repair (HRR) of DNA double-strand breaks. Hence, BRCA-deficient cases have a distinctive genomic aberration profile that makes them particularly susceptible to certain treatments, such as platinum-based chemotherapy regimens and PARP inhibitors (PARPi; refs. 3, 15). However, patients with *BRCA*-PV–associated breast cancer can develop resistance to DNA-damaging therapies through the appearance of reversion mutations, which restore the reading frame and can recover at least partial BRCA function (16, 17). Large genomic rearrangements, referred hereafter as copy-number variants (CNV), account for 10% to 40% of germline *BRCA1* and 3% of germline *BRCA2* PVs (18, 19). Pathogenic small insertion/deletion or base substitution variants that cause early protein truncation and CNVs both cause the loss of critical functional domains of BRCA proteins (20). However, with large segments of the gene missing, we presume that CNVs are less susceptible to reversion mutations. Thus, we hypothesize that carriers of *BRCA* CNVs with TNBC may have an improved survival after treatment compared with carriers of other *BRCA* PVs secondary to the inability of restoring BRCA expression.

We previously described a young Mexican TNBC cohort where the *BRCA1* ex9–12del CNV (also known as the Mexican *BRCA1* founder mutation) represented 40% of all germline *BRCA* PVs (21). Of note, TNBC represents a substantial proportion of breast cancer cases diagnosed in Mexico (16%–23%; refs. 22, 23), and is particularly prevalent in cases diagnosed at a young age (<50 years) and in those with germline *BRCA* PVs (4). The aim of this study is to compare the survival of young patients with TNBC with the *BRCA1* ex9–12del CNV, other *BRCA* PVs (small insertion/deletion or base substitution variants), and noncarriers to explore the influence of the *BRCA* genotype on survival.

## Materials and Methods

### Patient Cohort

A cohort of Mexican women diagnosed with TNBC at 50 years of age or younger between January 2006 and January 2012 that received treatment at the National Cancer Institute (INCan) in Mexico City has been described previously (21). To be eligible for this analysis, ER- and PR-negative status (<1% nuclear staining by IHC) and HER2 overexpression status (0 or 1+ by IHC or 2+ by IHC but demonstrated not to be amplified with FISH) was ascertained from pathology reports of tumor tissue. The patients provided written informed consent in accordance with recognized ethical guidelines (Declaration of Helsinki; U.S. Common rule), and the studies were approved by the INCan institutional review board.

### Screening for BRCA Germline PVs

As described previously (21), a total of 190 patients were analyzed for *BRCA* germline PVs using the HISPANEL assay, which screens for 114 indels and point *BRCA* PVs described as prevalent in Hispanic patients with breast cancer by five multiplex reactions on the Sequenom® (San Diego, CA) MassARRAY platform (matrix-assisted laser desorption/ionization—time-of-flight mass spectrometry) and includes a three-primer PCR assay for the *BRCA1* ex9–12del CNV (20).

### Clinicopathologic Data Collection

Electronic medical records from INCan's digital database were retrospectively reviewed up to December 31, 2019. The data collected for the 190 *BRCA*-characterized patients included patient age at diagnosis, clinical and/or pathologic TNM staging (according to the 7th edition of the American Joint Committee on Cancer Staging System), chemotherapy regimen used, histologic grade, mutational status, disease recurrence, second primary malignancies, and vital status. OS was defined as the time elapsed from histologic diagnosis of breast cancer until death by any cause and RFS was defined as the time elapsed from diagnosis until disease recurrence or death by any cause.

As the objective of this study was to compare OS and RFS rates between carriers of *BRCA1* ex9–12del, carriers of other *BRCA* PVs, and patients without a detectable *BRCA* PV, patients with *in situ* or stage IV disease were excluded from this analysis. Ultimately, 10 patients were excluded from this study: unspecified disease stage (*n* = 2), *in situ* disease (*n* = 1), and stage IV at diagnosis (*n* = 7), resulting in a total of 180 patients with *BRCA*-characterized TNBC included in the statistical analysis (Supplementary Fig. S1).

### Statistical Analysis

Statistical analyses were carried out using STATA version 13.0 software (StataCorp), R 4.0.2 (R Core Team 2020), and SAS9.4 packages. The patients were grouped according to *BRCA* mutational status. Descriptive statistics were undertaken using frequency and proportions for categorical variables and median and range for quantitative variables. Mann–Whitney *U*, *χ*^2^, and Fisher exact tests were used for exploring differences according to group category, as appropriate. OS and RFS were calculated using the Kaplan–Meier method. The log-rank test was used for survival comparisons between groups, and the exact calculation method was applied for comparisons involving zero events. Cox regression analyses were carried out to estimate the survival HRs associated with the class of *BRCA* germline PV. Noting that HR is undefined with zero events in one group, we reported survival and a confidence interval (CI) at a fixed time (e.g., 10 years), although with statistics from the log-rank test. The Beta Product Confidence Procedure (BPCP) was used to calculate the confidence intervals at specific timepoints if no survival failure events were observed in a group category. The BPCP method provides conservative confidence bounds, despite limited sample size. Statistical significance was set at a two-sided *P* value of < 0.05. No adjustment for multiple hypothesis testing was performed in the context of this retrospective analysis.

## Results

### Patient Demographics and Clinical Characteristics

A total of 180 patients with TNBC aged ≤50 years at diagnosis were included in this study, of which 43 patients (24%) were *BRCA* PV carriers. Of these, 17 (40%) were found to harbor the *BRCA1* ex9–12del CNV, while 26 (60%) had other *BRCA* PVs (Table 1). The median follow-up from breast cancer diagnosis for the entire cohort was 10.3 years (95% CI, 9.5–10.8). As shown in Supplementary Table S1, no statistical differences of median follow-up were found according to mutational status (median follow-up of 10.7 years for *BRCA* CNV carriers, 10.1 years for other *BRCA* PV carriers, and 10.3 years for noncarriers).

**TABLE 1:** *BRCA* PVs identified in this cohort of patients with TNBC.

Gene	Exon(s)	BIC variant	HGVS variant	*n*
*BRCA1*	9–12	ex9–12del	c.548-?_4185+?del	17
*BRCA1*	11	943ins10	c.815_824dup	5
*BRCA1*	5	330A>G (R71G)	c.211A > G	4
*BRCA1*	11	2925del4	c.2806_2809del	4
*BRCA1*	13	4446C>T (R1443X)	c.4327C > T	3
*BRCA1*	2	185delAG	c.66_67del	2
*BRCA1*	11	3878delTA	c.3759_3760del	2
*BRCA1*	11	3717C>T (Q1200X)	c.3598C > T	1
*BRCA1*	11	2415delAG	c.2292_2293del	1
*BRCA1*	18	5242C>A (A1708E)	c.5123C > A	1
*BRCA1*	11	1979delT	c.1860del	1
*BRCA1*	?	IVS20+1delG	c.5277+1del	1
*BRCA2*	11	2452C > T (Q742X)	c.2224C > T	1

Abbreviations: BIC, Breast Cancer Information Core; HGVS, Human Genome Variation Society.

Clinical characteristics are summarized in Table 2. *BRCA* PV carriers were diagnosed with breast cancer at a younger age than noncarriers (38 vs. 43 years; *P* < 0.001), with no observed difference between carriers of CNVs and other PVs. There were no significant differences observed between groups with respect to clinical stage or histologic grade. However, *BRCA* CNV carriers had a tendency toward negative lymph node status at diagnosis [10/16 (62.5%) CNV carriers with negative lymph node involvement vs. 9/25 (36%) other PV carriers vs. 43/136 (32%) noncarriers; three-group exact *χ*^2^ test *P* = 0.049]. *BRCA* PV carriers had a higher prevalence of bilateral breast cancer compared with noncarriers (19% vs. 4%; *P* = 0.003), with a nonsignificant excess of bilateral breast cancer in *BRCA* CNV carriers compared with the other PV group (24% vs. 15%; *P* = 0.692).

**TABLE 2:** Select clinicopathologic characteristics of included TNBC cases.

	*BRCA* mutation carriers					
	All *BRCA* PV carriers	*BRCA* CNV carriers^a^	Other *BRCA* PVs^b^	*P* ^c^	Noncarriers	*P* ^d^
*n*	43	17	26	—	137	—
Age at diagnosis						
Median (range)	38 (23–50)	39 (28–50)	37.5 (23–48)	0.784	43 (25–50)	**<0.001**
Missing	0	0	0	—	1 (1%)	
Bilateral BC	8 (19%)	4 (24%)	4 (15%)	0.692	5 (4%)	**0.003**
Stage						—
I	5 (12%)	2 (12%)	3 (12%)	0.326	8 (6%)	0.277
II	22 (51%)	11 (65%)	11 (42%)		63 (46%)	
III	16 (37%)	4 (24%)	12 (46%)		66 (48%)	
Nodal status						
Positive	22 (51%)	6 (35%)	16 (62%)	0.120	93 (68%)	0.095
Negative	19 (44%)	10 (59%)	9 (35%)		43 (31%)	
Missing	2 (5%)	1 (6%)	1 (4%)	—	1 (1%)	—
Histologic grade						
1	0	0	0	>0.999	4 (3%)	0.286
2	2 (5%)	1 (6%)	1 (4%)		13 (10%)	
3	40 (93%)	16 (94%)	24 (92%)		114 (83%)	
Missing	1 (2%)	0	1 (4%)	—	6 (4%)	—
Mastectomy						
Total	39 (91%)	16 (94%)	23 (89%)	0.691	117 (85%)	0.662
Partial	3 (7%)	1 (6%)	2 (8%)		14 (10%)	
Not performed	1 (2%)	0	1 (4%)		6 (4%)	
Chemotherapy						
Neoadjuvant	17 (40%)	7 (41%)	10 (39%)	0.947	67 (49%)	0.602
Adjuvant	17 (40%)	6 (35%)	11 (42%)		46 (34%)	
Both	9 (21%)	4 (24%)	5 (19%)		21 (15%)	
None	0	0	0		1 (1%)	
Missing	0	0	0	—	2 (2%)	—
Pathologic complete response^e^						
Yes	15 (58%)	7 (64%)	8 (53%)	>0.99	39 (44%)	0.362
No	10 (39%)	4 (36%)	6 (40%)		44 (50%)	
Missing	1 (4%)	0	1 (7%)	—	5 (6%)	—
Treatment with platinum-based chemotherapy						
Yes	23 (53%)	7 (41%)	16 (62%)	0.225	62 (45%)	0.484
No	20 (47%)	10 (59%)	10 (38%)		72 (53%)	
Missing	0	0	0	—	3 (2%)	—
Radiotherapy						
Yes	30 (70%)	12 (71%)	18 (69%)	>0.99	101 (74%)	0.695
No	13 (30%)	5 (29%)	5 (29%)		36 (26%)	
Risk-reducing surgeries						
Contralateral mastectomy^f^	14 (33%)	5 (29%)	9 (35%)	>0.99	2 (2%)	**<0.001**
Salpingo-oophorectomy	17 (40%)	8 (47%)	9 (35%)	0.528	0	**<0.001**
Second primary malignancy						
Any site	11 (36%)	7 (41%)	4 (15%)	0.080	10 (7%)	**0.002**
Breast	7 (64%)	4 (57%)	3 (75%)	—	5 (50%)	**—**
Ovarian	3 (27%)	2 (29%)	1 (25%)	—	1 (10%)	**—**
Thyroid	0	0	0	—	2 (20%)	**—**
Other^g^	1 (9%)	1 (14%)	0	—	2 (20%)	**—**

Abbreviation: BC, breast cancer.

^a^All *BRCA* CNV were *BRCA1* ex9–12del (Mexican founder mutation).

^b^An indel/point PV was identified in *BRCA1* in 25 patients and in *BRCA2* in one patient.

^c^ *P* value comparing *BRCA* CNV carriers versus other *BRCA* PVs.

^d^ *P* value comparing All *BRCA* PV carriers versus noncarriers.

^e^Only patients that received neoadjuvant treatment. Missing values are patients who did not undergo surgery (refused the procedure or died before the surgery was performed).

^f^Analysis restricted to contralateral mastectomy in patients without bilateral breast cancer (wherein the procedure is therapeutic).

^g^Other sites of second primary malignancies were cervical *in situ* cancer, vulvar *in situ* cancer, and Hodgkin lymphoma diagnosed in a single noncarrier of *BRCA* PVs, chondrosarcoma in another noncarrier patient, and bladder cancer in a *BRCA* Mexican founder mutation carrier.

Regarding general treatment strategies, no statistically significant differences were observed according to mutational status. Overall, 47% were treated with platinum-based chemotherapy regimens and none were treated with PARPi.

A total of 21 patients presented with a second primary malignancy during the follow-up period. In the *BRCA* CNV carrier group, second primaries were identified in the breast, ovaries, and bladder, while *BRCA* point mutation carriers were diagnosed with second primaries only in the contralateral breast and ovaries (Supplementary Table S2). *BRCA* PV carriers experienced a higher rate of second primary malignancies than noncarriers (36% vs. 7%; *P* = 0.002), with patients in the *BRCA* CNV group demonstrating a greater tendency, albeit nonsignificant, toward being diagnosed with a second cancer compared with other PV carriers (41% vs. 15%; *P* = 0.080).

### Survival Estimates

A total of 28 patients died during the follow-up period. The absence of deaths in the *BRCA* CNV group during the follow-up period is noteworthy. In contrast, 19% of carriers of other *BRCA* PVs and 17% of noncarriers experienced death. Of note, all deaths documented were attributed to the primary breast malignancy. Survival rates at 5 and 10 years are shown in Table 3.

**TABLE 3:** Survival outcomes at 5 and 10 years according to BRCA status.

	*BRCA* PVs carriers			
Outcome	All *BRCA* PV carriers	*BRCA* CNV carriers	Other *BRCA* PVs carriers	Noncarriers of *BRCA* PVs
OS				
At 5 years	90.7% (77.1–96.4%)	100% (80.5–100%)	84.6% (64.0–93.9%)	90.4% (84.1–94.3%)
At 10 years	87.3% (71.8–94.6%)	100% (73.5–100%)	78.6% (55.0–90.7%)	84.7% (76.8–90.1%)
RFS				
At 5 years	86.1% (71.6–93.5%)	94.1% (65.0–99.2%)	80.8% (59.8–91.5%)	83.8% (76.5–89.1%)
At 10 years	83.6 (68.6–91.8%)	87.8% (59.5–96.8%)	80.8% (59.8–91.5%)	77.8% (69.6–84.1%)

NOTE: Data are shown as Kaplan–Meier estimate (95% CI).

**FIGURE 1:**
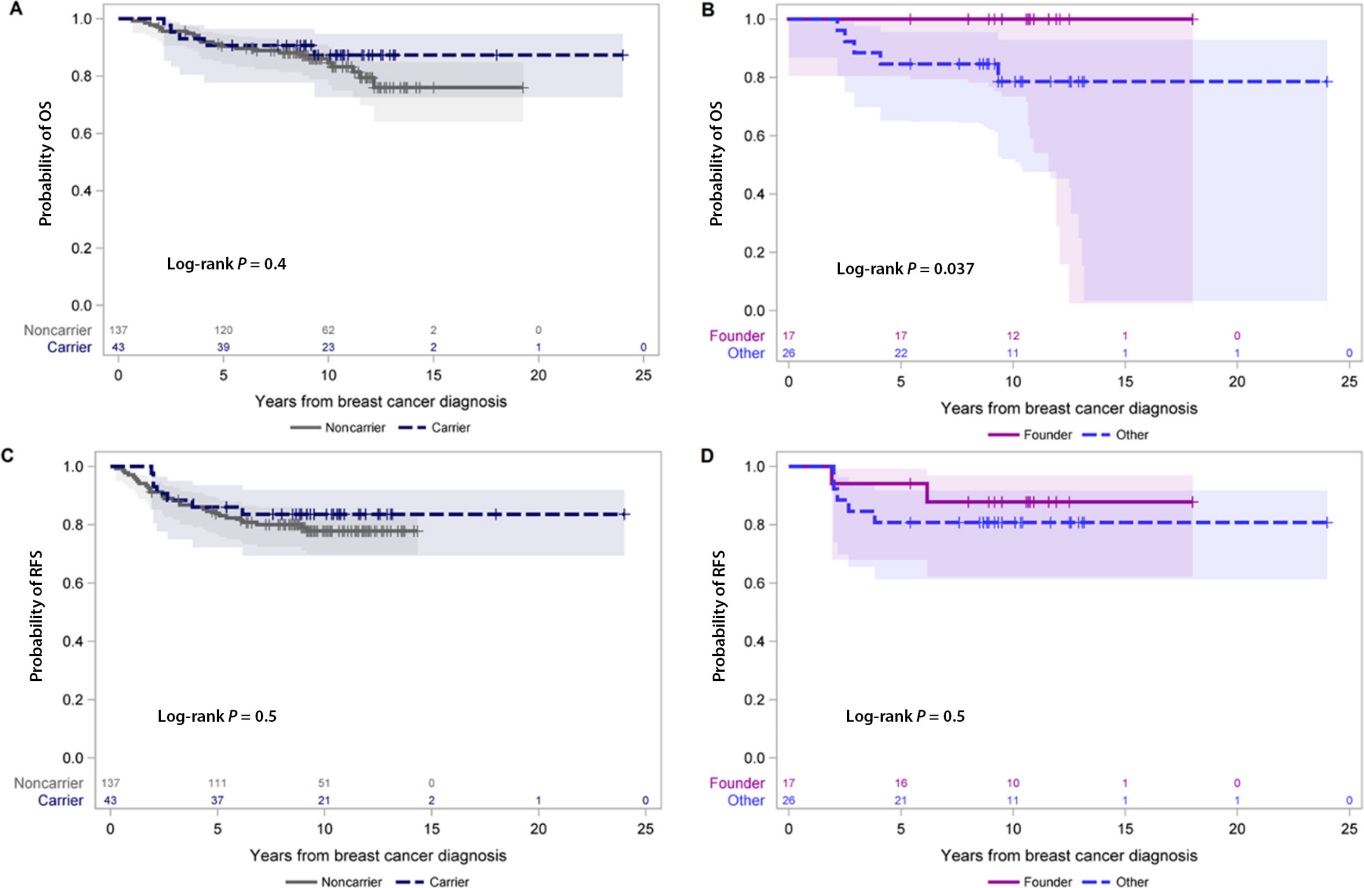
**A,** Survival estimates according to *BRCA* status OS of *BRCA* germline PVs carriers versus noncarriers. **B,** OS of carriers of *BRCA1* ex9–12del CNV versus carriers of other *BRCA* PVs. **C,** RFS of *BRCA* germline PVs carriers versus noncarriers. **D,** RFS of carriers of *BRCA1* ex9–12del CNV versus carriers of other *BRCA* PVs.

OS was 86.3% (95% CI, 80.0–90.7) for the whole cohort at ten years after diagnosis of TNBC. Overall, no statistically significant differences were observed in OS between *BRCA* PV carriers and noncarriers [HR, 0.66; 95% CI, 0.25–1.74). However, when comparing *BRCA* CNV carriers versus carriers of other BRCA PVs (Fig. 1A and B), OS was significantly higher in the former (100% vs. 78.6% at 10 years; log-rank *P* value of the Kaplan–Meier model = 0.037). OS rates of *BRCA* CNVs were also numerically superior to noncarriers, although not reaching statistical significance (100% vs. 84.7% at 10 years; log-rank *P* = 0.06).

The survival advantage observed in the carriers of *BRCA* CNVs was maintained after exclusion of the one patient in the group of carriers of other *BRCA* PVs that had a PV in *BRCA2* (100% vs. 77.5% at 10 years; two-sided log-rank *P* value of the Kaplan–Meier model = 0.030). Of note, node-negative patients had 0 of 10 registered deaths in CNV group versus 1 of 9 in other PV group, while node-positive patients had 0 of 6 deaths documented in CNV group and 4 of 16 deaths in the other PV group.

In total, 34 patients experienced disease recurrence. As shown in Table 2, all recurrences documented in *BRCA* PV carriers and the majority of those experienced by noncarriers were classified as distant disease. Overall, the RFS rate for the whole cohort was 79.2% (95% CI, 72.3–84.6) at ten years after diagnosis of TNBC. No statistically significant differences were observed in RFS between groups (Table 3; Fig. 1C and D).

## Discussion

In this study, we explored the survival outcomes of a cohort of *BRCA*-characterized women with TNBC aged ≤50 years at diagnosis. Notably, OS rates did not differ significantly between carriers of *BRCA* PVs and noncarriers. However, when examining subgroups according to *BRCA* variant type, a survival advantage was observed in the *BRCA* CNV carrier group. Specifically, the carriers of the *BRCA1* ex9–12del CNV in our cohort had a 100% OS rate at 10 years, which was numerically superior to the 10-year OS observed among carriers of other *BRCA* PVs and noncarriers (78.6% and 84.7%, respectively). We believe that the findings of our study have important clinical prognostic and treatment implications for patients with breast cancer who carry a *BRCA* PV as they suggest that genotype can exert a differential impact on survival. Further studies confirming the observed difference are needed.

The Mexican *BRCA1* founder CNV (*BRCA1* ex9–12del) is one of the most frequently reported population-specific CNVs (24). It is characterized by a deletion of 14.7 kilobases, which results in the loss of multiple functional protein domains and premature BRCA truncation. Specifically, the *BRCA1* ex9–12del CNV results in the complete loss of the p53, pRB, Rad50, Rad51, NLS1, and NLS2 interacting domains, and partial loss of the ERα domain (20). This aberration was first reported in 2007 after it was detected in 3.8% of unrelated Hispanic American families that had a personal history of breast cancer or ovarian cancer (20). In our referral center, it has been reported that this PV accounts for 35% of BRCA-associated ovarian cancer cases and 29% of BRCA-associated breast cancer cases (24). The remarkable frequency of the Mexican *BRCA1* founder mutation, particularly in women originating from Central and Southern Mexico, constitutes a regional public health problem. Given its high prevalence in our setting, we employed the *BRCA1* ex9–12del genotype to analyze if CNVs have a different prognostic impact than point mutations in the same gene.

Previous studies have raised the possibility of *BRCA* PVs having a different prognostic impact based on factors other than their germline presence. For example, a meta-analysis by Xie and colleagues showed that, although the presence of *BRCA1* PVs had no correlation with prognosis in patients with breast cancer, *BRCA1* promoter methylation was associated with worse survival outcomes (10). Hollis and colleagues summarized the evidence regarding the implications of different germline *BRCA* PVs in ovarian cancer and concluded that aberrations at particular *BRCA1* sites could confer differential sensitivity to platinum-based chemotherapy and PARPi (14). Furthermore, a study of patients with ovarian cancer showed that the presence of *BRCA2* PVs in the ovarian cancer cluster region was associated with a better RFS than those in breast cancer cluster regions and not-related risk regions (25). Drost and colleagues investigated mice carrying the *BRCA*1185stop and *BRCA*15382stop alleles (which mimic the most common human *BRCA1* founder mutations) and showed that the *BRCA*1185stop mice responded markedly worse to HRR deficiency–targeting therapies (13). Together, these studies suggest that not all *BRCA* PVs have the same impact on tumor phenotype, treatment response, or survival outcomes.

Reversion mutations are secondary alterations in a mutant allele that restore partial or complete protein functionality by reverting an initial frameshift mutation into an in-frame internal deletion (26). In *BRCA*-associated breast tumors, the appearance of acquired somatic reversion mutations has been described as a potent oncogenic event to resist unwanted DNA damage though the restoration of BRCA expression (27). Thus, the development of reversion mutations in *BRCA* PV carriers with breast cancer could have an adverse prognostic impact by limiting the effectiveness of DNA-damaging treatment strategies such as platinum-based chemotherapy regimens. The clinical significance of reversion mutations was illustrated by Lin and colleagues in a study where the absence of *BRCA* reversion mutations in circulating cell-free DNA of patients with ovarian cancer was associated with a significantly longer rucaparib progression-free survival (16).

Recently, it was reported in a small study that patients with ovarian cancer harboring the Mexican *BRCA1* founder CNVs had a better RFS than those with other types of *BRCA1* PVs (25). However, the prognostic role of CNV such as the Mexican *BRCA1* founder mutation on BC had not been previously reported. We postulate that the significantly enhanced OS in the *BRCA1* ex9–12del PV carrier group found in our cohort occurs secondary to the inability of reversion mutations to restore the vast deletion associated with this CNV. We believe that patients with TNBC who carry this PV could tend toward sustained responses to DNA-damaging therapies (such as chemotherapy regimens based on the use of platinum or anthracyclines plus alkylating agents; refs. 28, 29), and this may also relate to trend in survival when comparing CNV carriers to noncarriers. However, the precise mechanisms underlying the observed differences among Mexican *BRCA1* founder mutation carriers and other *BRCA* PVs are presently unknown and warrant further study.

Our study has several limitations. Full sequencing for *BRCA* PVs was not undertaken and was limited to those included in the HISPANEL assay (sensitivity 77%; ref. 24). Therefore, some of the patients classified as noncarriers of *BRCA* mutations could carry PVs that were not detected using our methodology. However, we believe any bias introduced by misattributed cases would not affect the survival analysis of CNV carriers versus other PV carriers. In addition, the HISPANEL assay does not identify PVs in other breast cancer susceptibility genes, which could have been present in some of our patients. Few panel genes (e.g., *PALB2*) have evidence for impact on survival or evidence for benefit from DNA-damaging therapies. Again, misattribution would not affect the survival advantage observed in CNV carriers compared with other PV carriers. The cohort selected for this analysis is limited by the small sample size and could be subject to survivorship bias as a median of 37.5 months had elapsed from breast cancer diagnosis to study inclusion. Therefore, patients with poor survival could have been disproportionately excluded, and this could be responsible in part for the relatively favorable outcomes for the entire study population. However, as all groups were subject to the same bias, we believe the study's subsequent prospective outcomes are still valid. Moreover, the follow-up after inclusion was long, and OS and RFS calculated from time of study accrual demonstrated the same tendencies toward improved survival in the carriers of *BRCA* CNV group. Notably, all deaths registered in *BRCA* PV carriers were attributable to breast cancer. Hence, breast cancer–specific survival was essentially equivalent to OS in *BRCA* carriers, and the differences we observed were not due to mortality from a second primary malignancy. Nonetheless, it is possible that the benefit observed in survival outcomes in CNV carriers was influenced by a lower prevalence of lymph node invasion and stage III disease. The only large CNV observed in our cohort was the Mexican *BRCA1* founder mutation, which limits the extrapolation of the prognostic impact of CNVs observed in this study to other *BRCA* CNVs. However, we suggest that any CNV with a comparably large deletion would limit the potential for reversion mutations and result in improved survival. Finally, due to the retrospective nature of this study, no adjustment for multiple hypothesis testing was performed. Hence, an inflation of type I error due to multiple testing cannot be excluded. Combined with the issues listed above, this underscores the need to interpret the results of this study as hypothesis-generating and independent validation is required. Despite these limitations, we believe that this analysis provides evidence supporting a survival impact from specific *BRCA* genotypes, with a plausible mechanistic underpinning.

In conclusion, this study demonstrates that the type of germline BRCA PV can influence survival after treatment for BC. We demonstrated that patients with TNBC with the *BRCA1* ex9–12del had statistically better OS than carriers of other *BRCA* PVs. This could have important prognostic and clinical implications. Future studies are needed to confirm these findings and test if the observed survival differences are due to different susceptibility to reversion mutations or other factors.

## Supplementary Material

Supplementary Data supplemental tables
