# Supplementary material for: Influence of Germline BRCA Genotype on the Survival of Patients with Triple-Negative Breast Cancer
Source: Cancer Res Commun. 2021 Dec 8;1(3):140–7. doi: 10.1158/2767-9764.CRC-21-0099 (PMC9307147; doi:10.1158/2767-9764.CRC-21-0099)
Supplement: Supplementary Data — supplemental tables [file crc-21-0099-s01.docx]

**Supplementary material**

Supplementary figure S1. Study flow diagram.


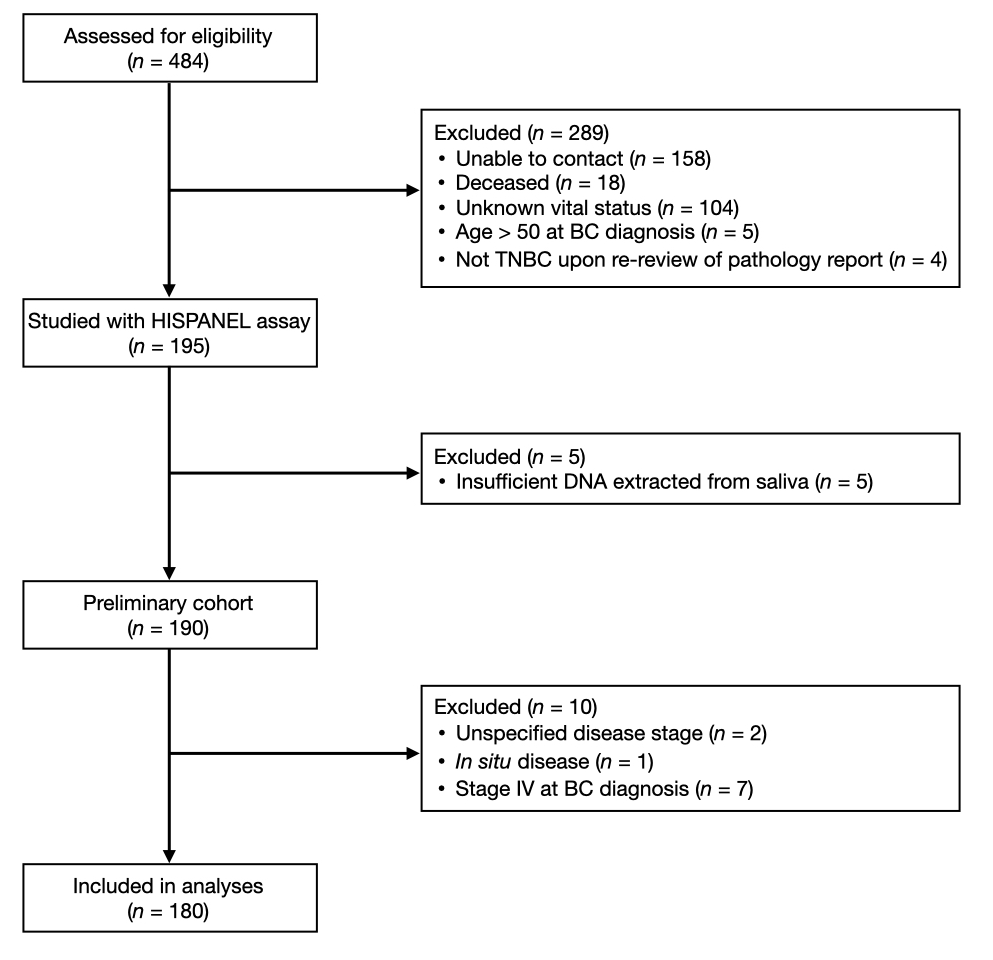


Supplementary table S1. Median follow-up according to group category

|  | *BRCA* mutation carriers | | | | Non-carriers | *p-*value*^‡^* |
| --- | --- | --- | --- | --- | --- | --- |
|  | **All *BRCA* PV carriers** | ***BRCA* CNV carriers** | ***Other BRCA* PVs** | ***p-*value^†^** |  |  |
| Median months elapsed from diagnosis to last contact (95%CI)* | 128 (112-139) | 128 (110-143) | 121 (106-150) | 0.698 | 123 (113-131) | 0.519 |
| Median months elapsed from diagnosis to study inclusion (95%CI)** | 38 (24-45) | 41 (21-52) | 30 (23-52) | 0.658 | 37 (30-46) | 0.724 |
| Median months elapsed from study inclusion to last contact (95%CI)* | 87 (85-89) | 87 (84-91) | 87 (82-89) | 0.339 | 86 (85-87) | 0.804 |

^†^ log-rank *p*-value comparing BRCA CNV carriers vs. other BRCA PVs
^‡^ log-rank *p*-value comparing All BRCA PV carriers vs. non-carriers

*Calculated using the reverse Kaplan-Meier method

**Calculated using the Kaplan-Meier method

Supplementary table S2. Stage distribution of ovarian and breast second primary malignancies according to mutational status.

| 2^nd^ Primary malignancy | CNV | Other PV | Non-carriers |
| --- | --- | --- | --- |
| Any site | 7 (41%) | 4 (15%) | 10 (7%) |
| Breast  *In situ*  Stage 1  Stage 2 | 1 (25%)  2 (50%)  1 (25%) | 0  2 (33%)  1 (33%) | 0  4 (80%)  1 (20%) |
| Ovarian  Stage 1  Stage 2  Stage 3  Stage 4  Missing | 0  0  2 (100%)  0  0 | 0  0  0  0  1 (100%) | 1 (100%)  0  0  0  0 |
| Other* | 1 (6%) | 0 | 4 (3%) |

*Other second primary malignancies were thyroid cancer, chondrosarcoma, and Hodgins’s lymphoma in non-carriers and bladder cancer in CNV carriers.
